# Elevated filling pressures but rare restrictive physiology in transthyretin amyloid cardiomyopathy: genotype-specific patterns and the role of left atrial strain

**DOI:** 10.1007/s10554-026-03667-z

**Published:** 2026-03-04

**Authors:** Fredrik Edbom, Ashwin Venkateshvaran, Sandra Arvidsson

**Affiliations:** 1 Department of Diagnostics and Intervention, Clinical Physiology, Umeå University, Umeå, Sweden; 2 Department of Clinical Sciences, Clinical Physiology, Lund University, Lund, Sweden

**Keywords:** Echocardiography, Transthyretin amyloid cardiomyopathy, Diastolic function, Elevated filling pressure, Restrictive filling pattern, Left atrial reservoir strain

## Abstract

Transthyretin amyloid cardiomyopathy (ATTR-CM) is associated with cardiac stiffness and diastolic dysfunction. While recommendations highlight restrictive filling as a common echocardiographic marker in ATTR-CM, a detailed characterization of diastolic dysfunction (LVDD) severity and differences in prevalence between ATTR-CM genotypes have not been explored. In a well-characterized ATTR-CM cohort, we investigated the prevalence, severity and genotype-specific distribution of LVDD and assessed the added value of left atrial reservoir strain (LASr) for its detection. Diastolic dysfunction was graded by transthoracic echocardiography in 100 patients with ATTR-CM (76 hereditary [ATTRv] and 24 wild-type (ATTRwt) according to current ASE/EACVI recommendations, with LASr incorporated for comparison. Restrictive filling was observed in only 22% of the cohort and was more common in in ATTRwt compared with ATTRv (59% vs. 14% respectively). Incorporating LASr into LVDD assessment did not change the proportion of patients with restrictive filling pressure but increased the proportion of patients identified with elevated filling pressure from 53% to 68% and reduced indeterminate cases from 13% to 3%. Restrictive filling in uncommon in ATTR-CM and varies by genotype, while incorporation of LASr markedly improves detection of elevated filling pressure. These findings underscore the need for a personalized approach and precision therapy tailored to genotypical differences and comorbidities.

**Supplementary Information:**

The online version contains supplementary material available at 10.1007/s10554-026-03667-z.

## Introduction

Diastolic dysfunction is a hallmark of cardiac amyloidosis (CA), an infiltrative cardiomyopathy characterized by the deposition of amyloid fibrils in the myocardium. Amyloid infiltration reduces LV compliance and impairs relaxation leading to diastolic dysfunction [1] and may progress to restrictive filling hemodynamics in later stages of the disease [2].

Transthoracic echocardiography (TTE) is used as a first-line screening tool to raise suspicion of CA [3], evaluate cardiac function and grade diastolic dysfunction. A recent echocardiographic supplement to expert consensus recommendations suggests that grade II or grade III left ventricular diastolic dysfunction (LVDD) is common in CA, indicating elevated left atrial pressures (LAP) and/or restrictive LV filling [4]. However, some studies report only mild diastolic abnormalities [5], whereas others describe a higher proportion of restrictive filling hemodynamics [6]. Transthyretin amyloid cardiomyopathy (ATTR-CM) is a heterogenous group, with hemodynamic profiles that vary based on genotype and disease progression [5, 7]. Comparative data on diastolic status between subgroups is lacking but may inform precision medicine approaches. Recent studies have suggested that incorporation of left atrial reservoir strain (LASr) improves diagnostic performance of current diastolic assessments [8]. However, this has not yet been systematically evaluated in ATTR-CM.

We sought to evaluate the severity of left ventricular diastolic dysfunction (LVDD) using conventional echocardiographic indices in a well-defined ATTR-CM cohort, to compare phenotypic presentation between hereditary (ATTRv) and wild-type (ATTRwt) subgroups, and to assess the incremental value of left atrial reservoir strain (LASr) for identifying elevated filling pressures.

## Method

### Patient population

We conducted a retrospective, observational study based on prospectively collected data from an existing database of 238 ATTR patients at Umeå University Hospital between 2006 and 2021. ATTR diagnosis was confirmed by genetic testing and tissue biopsy. Cardiac involvement was assessed using 99mTc-labelled 3,3-diphosphono-1,2-propanodicarboxylic acid (DPD) bone scintigraphy, available for the vast majority of participants.

We included patients with echocardiographic signs of hypertrophy, defined as interventricular septum thickness (IVST) > 12 mm in women and > 14 mm in men, together with cardiac involvement based on positive 99mTc-DPD -bone scintigraphy (grade II and III) when available.

Patients with paroxysmal atrial fibrillation (pxAF) were included, provided they were in sinus rhythm at the time of the echocardiographic examination. Patients with persistent or permanent atrial fibrillation (AF), or who were in AF rhythm during the examination, were excluded. Additional exclusion criteria were moderate or severe valvular disease, heart failure due to prior myocardial infarction, poor image quality, or insufficient echocardiographic data for comprehensive diastolic assessment, such as patients with only ≤ 1 echocardiographic diastolic parameter available.

NT-proBNP levels obtained within three months of the echocardiographic exam date was accepted. Diagnoses of hypertension, AF or pxAF, diabetes, pacemaker implantation, and CHD were extracted from medical records.

The study complies with the Declaration of Helsinki and was approved by the regional ethics review board in Umeå (DNR 2016/435-31 M with supplementary application DNR 2018-418-32 M). All the participants provided written informed consent.

### Echocardiography

Comprehensive 2-D and Doppler echocardiographic examinations were performed on GE vivid E7, Vivid E9 and Vivid E95 systems (GE Medical Systems, Horten Norway) equipped with an adult 1.5–4.3 MHz phased array transducer with the patient in the left lateral decubitus position. All cine images were captured using a frame rate of 50–70 fps. Images were digitally stored in DICOM-format and exported. Analysis and measurements were performed retrospectively and offline using EchoPAC software (EchoPAC version 204, GE Vingmed Ultrasound AS, USA) by an experienced operator (FE) blinded to clinical data.

Measurements and chamber quantification were performed in accordance with current recommendations [9]; LV end-diastolic diameter (LVEDD, LV end-systolic diameter (LVESD), IVST and posterior wall thickness (PWT) were measured in two-dimensional parasternal long-axis images. LV mass was calculated using the Devereux formula. Left atrial volume was obtained by the modified Simpson’s biplane method, to trace the left atrial cavity in apical 4- and 2-chamber view and indexed to BSA (LAVI). The biplane method was also used when tracing the LV cavity in apical 4- and 2-chamber view for LV ejection fraction (LVEF) estimation.

Diastolic function was assessed according to current recommendations [10] using indices of mitral inflow LV myocardial relaxation and tricuspid regurgitation (TR). Early (E) and late (A) mitral inflow velocities were obtained using a 1–3 mm sample volume placed at the tip of the mitral leaflets. E/A ratio and early diastolic deceleration time (DT) were estimated using an average of three consecutive beats. Mitral annular early diastolic myocardial tissue velocities (e’) were obtained from the septal and lateral LV annulus using tissue Doppler imaging (TDI), aligned with the axis of movement of the annular plane. An average of septal and lateral e’ was used to calculate E/e’ ratio.

Tricuspid regurgitation (TR) peak velocity was measured employing continuous-wave (CW) Doppler recordings with the cursor aligned with the tricuspid regurgitation jet in the appropriate view. Grading of LVDD by recommended two-dimensional and Doppler methods was performed in accordance with the 2016 ASE/EACVI algorithm for myocardial disease [10]. LVDD grade I corresponds to normal LAP, and grade II and III to elevated LAP, thus grade II and III were pooled as ‘elevated LAP’. The presence of restrictive filling pattern was defined as LVDD grade III.

Mean LV global longitudinal strain (LV GLS) was obtained by speckle-tracking using the apical 4-, 3- and 2-chamber views. To evaluate LASr, longitudinal peak atrial strain was assessed in a similar approach from apical 4- and 2-chamber views. Care was taken to adjust the region of interest to match both LV myocardial thickness and the thin LA wall. Manual adjustments were made to ensure optimal tracking throughout the cardiac cycle. If overall tracking quality was deemed inadequate despite adjustments, the measurement was discarded from further analysis. The R-wave in the QRS complex was used as the zero point.

In keeping with the latest expert recommendations and for comparative purposes, LVDD grading was also performed by replacing TR peak velocity with LASr for, with a cutoff < 18% representing an abnormal finding.

### Statistical analysis

Continuous variables were expressed as median (IQR) according to distribution and categorical variables as counts (n) and percentages. Normality of data was asserted using Shapiro-Wilk test. Comparisons of demographic, laboratory, and echocardiographic variables between ATTR subgroups and for comparison between normal (LVDD grade I) and elevated LAP (LVDD grade II and III) groups were done by Mann-Whitney U test. Effect size reported as the Hodges-Lehman estimator of the median difference with 95% confidence interval (CI).

LVDD grade was treated as an ordinal categorical variable. Differences in the distribution of LVDD grades between ATTR genotypes were assessed using contingency table analysis. Overall distributions were compared using the Fisher’s exact test, and ordered trends across LVDD grades were evaluated using the linear-by-linear association test. Effect size was expressed as risk ratio with 95% CI.

Given the limited number of outcome events and the exploratory nature of adjusted analyses, covariate selection was restricted to a minimal, prespecified set of clinically relevant confounders to avoid overfitting and overadjustment. Variables considered downstream markers of disease severity were not included simultaneously. A p-value < 0.05 was considered statistically significant.

Statistical analyses were performed using IBM SPSS Statistics version 29 (IBM Corp., Armonk, NY, USA) and R version 2026.01.0 (R Foundation for Statistical Computing, Vienna, Austria). Figures were generated using Python.

## Results

### Study population

**Fig. 1:**
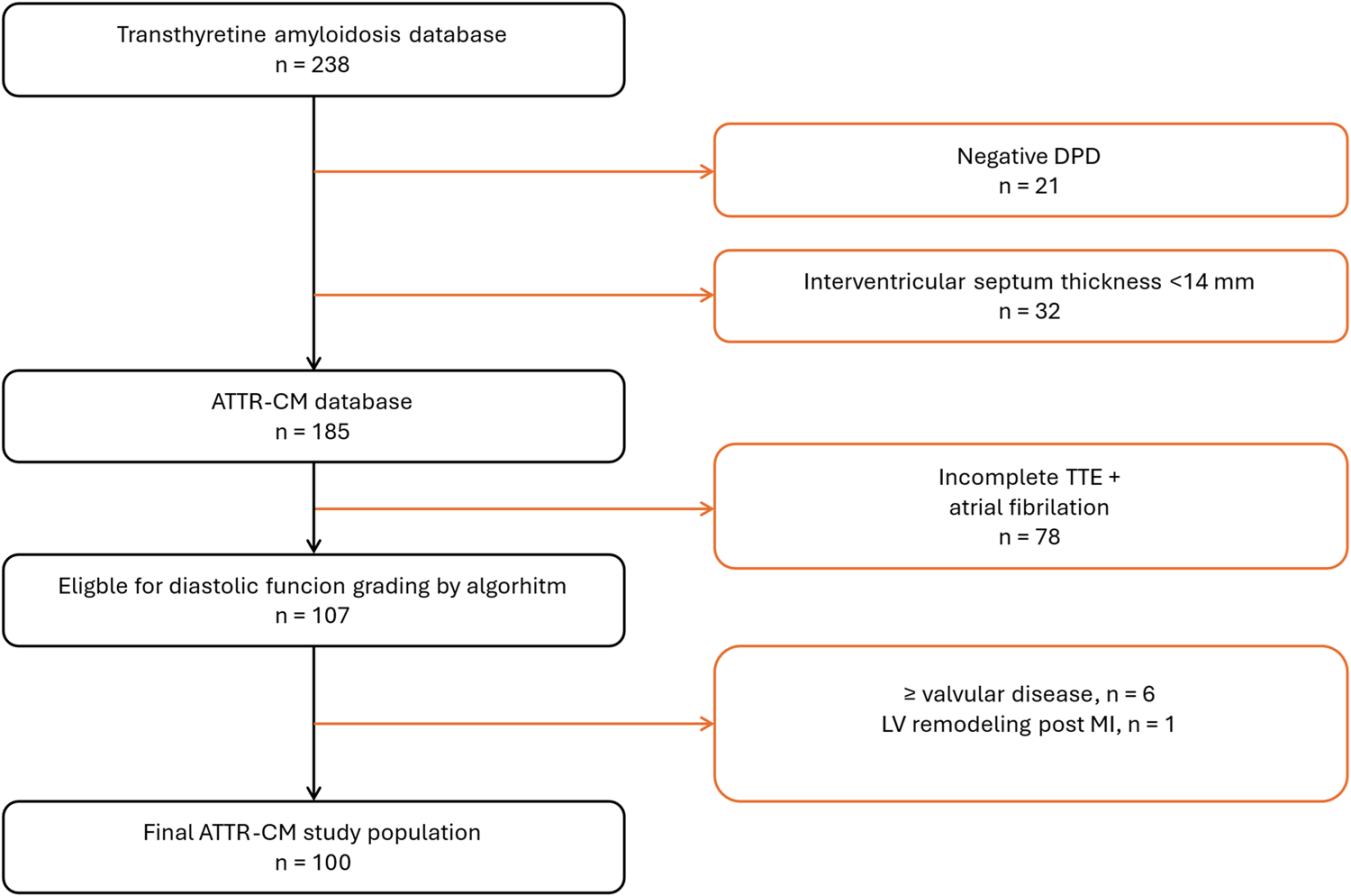
Flow diagram of how the study participants met with the inclusion and exclusion criteria

Of 238 ATTR patients, 185 displayed cardiac involvement according to definition with signs of increased myocardial thickness. After excluding patients with permanent AF, poor image quality or inadequate Doppler information for diastolic assessment (*n* = 78), significant valvular disease (*n* = 6) and LV remodeling post MI (*n* = 1), 100 patients (27% females) with ATTR-CM were included in the final analysis (Fig. 1).

Positive DPD grades II and III were available in *n* = 93 (93%) of the participants included in the study population. Of these, echocardiographic data was obtained from examinations obtained within < 12 months from DPD-date in *n* = 76 (82%) cases, 12–24 months after DPD in *n* = 14 (15%), > 24 after DPD *n* = 5 (5%), with the longest follow-up occurring approximately 7.5 years after DPD-date (Supplementary Materials Figure S1).

Clinical characteristics for all participants as well as grouped by ATTR subtypes are listed in Table 1.

**Table 1:** Clinical characteristics in total ATTR-CM and subgroups

	All participants (*n* = 100)	ATTRv (*n* = 76)	ATTRwt (*n* = 24)	Effect size (95% CI)	*p*-value ATTRv vs. ATTRwt
Age, years	72.5 (12)	70 (11)	79 (12)	-8 (-11 to -4)	< 0.001
Sex, female gender, n (%)	27 (27.0)	23 (29.1)	5 (17.9)	RR 0.55 (0.21–1.43)	0.216
BSA, m^2^	1.89 (0.27)	1.88 (0.30)	1.89 (0.26)	-0.07 (-0.18–0.04)	0.214
Type 2 diabetes	5 (5.0)	3 (3.9)	2 (8.3)	RR 2.1 (0.37–11.9)	0.591
Pacemaker, n (%)	10 (10.0)	8 (10.1)	2 (7.1)	RR 0.79 (0.18–3.48)	1.0
Essential hypertension, n (%)	32 (32.0)	22 (27.8)	7 (22.6)	RR 0.886 (0.439–1.788)	0.806
PX atrial fibrillation, n (%)	28 (28.0)	11 (13.9)	17 (54.9)	**RR 3.17 (1.829–5.484)**	**< 0.001**
CHD, n (%)	18 (18.0)	9 (11.4)	10 (32.3)	RR 2.01 (0.880–4.617)	0.129
NT-ProBNP, µg/L	1165 (1924)	1126 (1964)	1165 (1636)	-474 (-896–53)	0.077
Genotype					
Ala45Gly		1 (1.3%)			-
Ala97Ser		1 (1.3%)			-
Glu54Leu		2 (2.6%)			-
His88Arg		5 (6.6%)			-
Thr60Ala		1 (1.3%)			-
Val122LIle		1 (1.3%)			-
Val30Met		65 (85.5%)			-

Data are presented as median (IQR) or n (%), as appropriate

Abbreviations: BSA, body surface area; CHD, coronary heart disease; NT-proBNP, N-terminal pro-B-type natriuretic peptide; Px, Paroxysmal

The study group consisted of 24 (24%) patients with ATTRwt and 76 (76%) patients with ATTRv, of which the vast majority presented with Val30Met genotype (85.5 of ATTRv). The median age for all ATTR-CM participants was 72. (IQR 12) years and 27% were females. The ATTRwt group was significantly older than the ATTRv group, with a median age difference of 8 years (95% CI − 11 to − 4; *p* < 0.001). In addition, a prior history of atrial fibrillation was more prevalent in ATTRwt, with paroxysmal AF observed in 28 patients (28.0%) overall, including 11 ATTRv patients (13.9%) and 17 ATTRwt patients (54.9%), corresponding to a relative risk of 3.17 (95% CI 1.83–5.48; *p* < 0.001).

### Assessment of diastolic dysfunction using ASE/EACVI recommendations in ATTR-CM

**Fig. 2:**
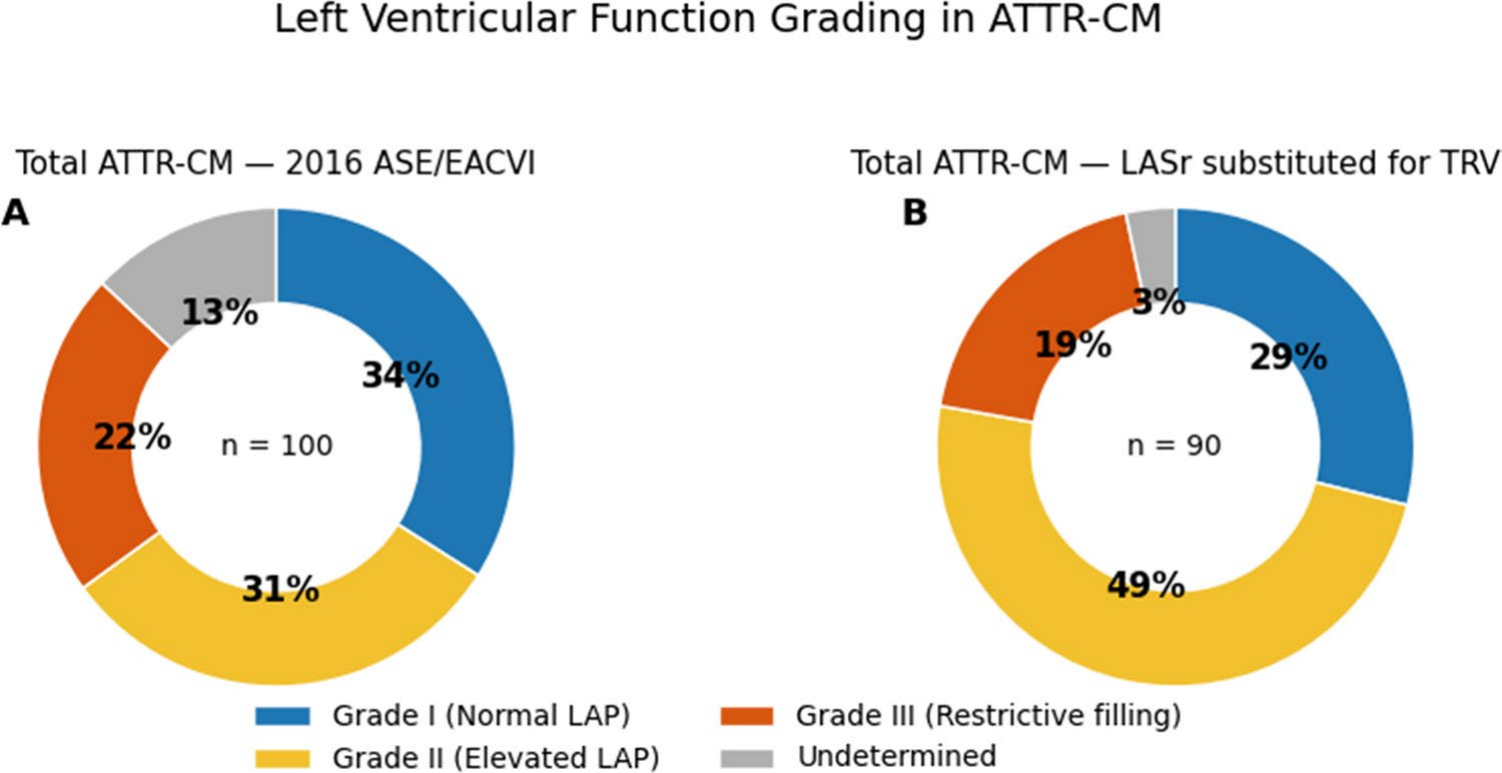
Left ventricular diastolic function grading displayed in total Transthyretin Amyloid Cardiomyopathy (ATTR-CM) study population, evaluated using (**A**) the 2016 ASE/EACVI diastolic function algorithm and (**B**) by substituting the tricuspid regurgitation velocity with left atrial reservoir strain (LASr)

In the total ATTR-CM population, diastolic dysfunction could be graded by the 2016 ASE/EACVI algorithm in 87% of patients, leaving 13% as Undetermined LAP (Fig. 2).

Thirty-four (34%) participants of the total study cohort presented with normal LAP. While the majority (53%) displayed elevated LAP (LVDD grade II and III), restrictive filling was limited to 22%. No grading was determined in 13% of the cases (Fig. 2). Among ATTR-CM participants who could be classified into LVDD grades I–III, the availability of diastolic function parameters was 74% for E/e′, 70% for LAVI, 70% for TRV, and 90% for LASr (Supplementary Table S1).

**Fig. 3:**
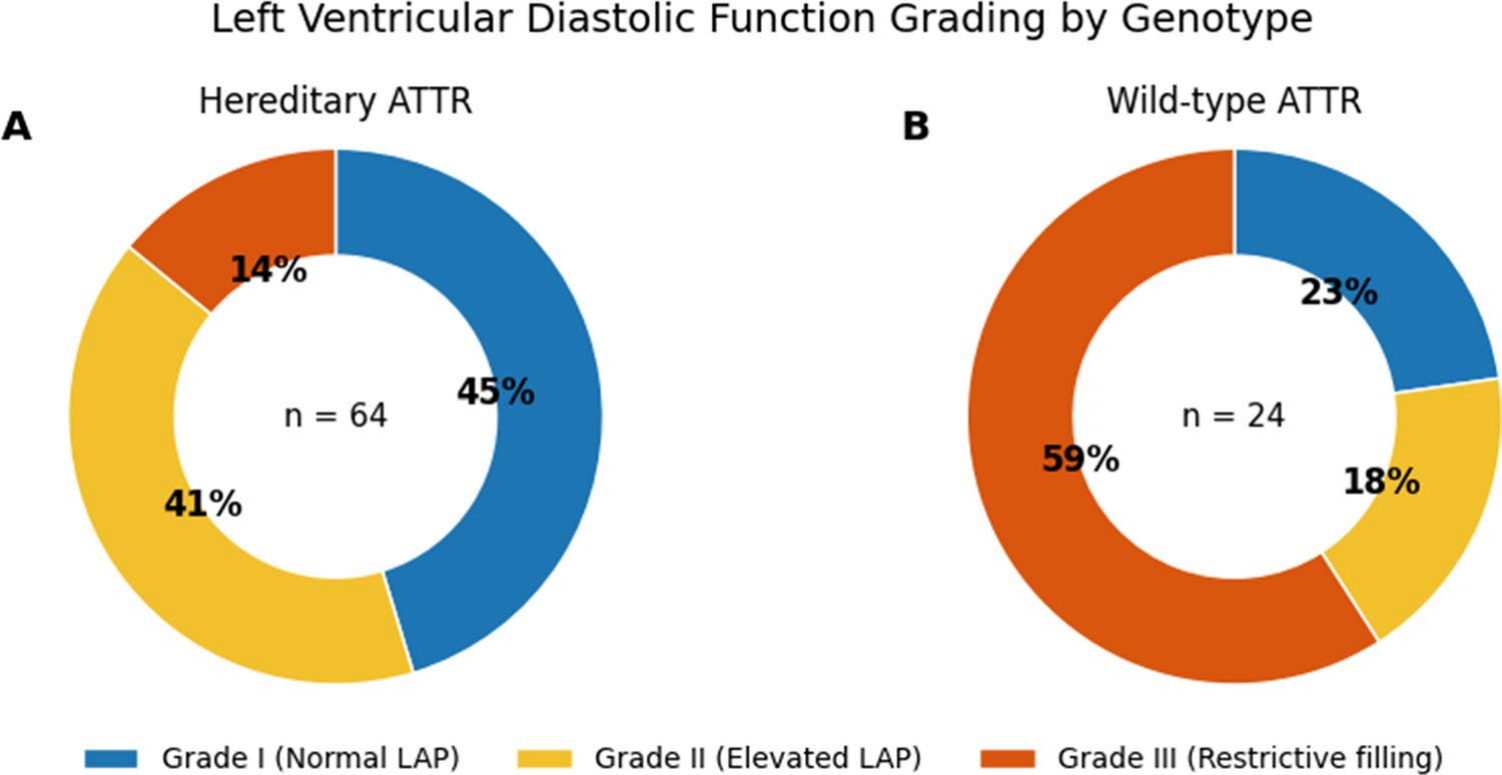
Proportions of left ventricular diastolic function grades in (**A**) hereditary ATTR and (**B**) wild-type ATTR, evaluated using the 2016 ASE/EACVI diastolic function algorithm

Excluding patients with undetermined LAP, ATTR-CM subgroups showed differential prevalence of LVDD severity (Fig. 3).

In ATTRv, 45% of patients displayed indices of normal LAP and **55%** indices of elevated LAP (Fig. 3). Interestingly, in ATTRwt, only 23% exhibited normal LAP, whereas 77% demonstrated elevated LAP.

The distribution of LVDD grades differed markedly between ATTR genotypes (*p* < 0.001). Restrictive filling (LVDD grade III) was substantially more common in ATTRwt than in ATTRv patients (59% vs. 14%). This corresponded to a fourfold higher prevalence of restrictive filling in ATTRwt indicating a significant shift toward more severe LVDD to grade III in ATTRwt (risk ratio 4.3, 95% CI 2.1 to 8.3, *p* < 0.001, Table 2).

**Table 2:** Echocardiographic variables in total ATTR-CM and subgroups

Variables	All participants (*n* = 100)	ATTRv (*n* = 76)	ATTRwt (*n* = 24)	Effect size (95% CI)	*p*-value (ATTRv vs. ATTRwt)
LVIDd, mm	44 (8)	43 (8)	44 (8)	0 (-3–2)	0.728
IVST, mm	17 (4)	17 (4)	18 (4)	-1 (-2–0)	0.086
LV mass, g	273 (87)	265 (87)	298 (69)		0.141
LAVI (mL/m^2^)	38.4 (13.6)	37.2 (13.3)	46.1 (13.9)	**-8.75 (-13.3 to -4.08)**	**0.001**
CO (L/min)	5.1 (1.9)	5.2 (2.1)	4.0 (2.0)	**0.75 (0.07–1.36)**	**0.028**
LVEF (%)	51 (12)	52 (12)	48 (9)	4 (0–8)	0.074
Normal LVEF ≥ 50% n, (%)	58 (58)	49 (62)	14 (45.)	RR 0.73 (0.45–1.17)	0.160
HFmrEF 41–49% n, (%)	29 (29)	21 (27)	12 (39)	RR 1.48 (0.78–2.83)	0.300
HFrEF < 41% n, (%)	12 (12)	8 (10)	5 (16)	RR 1.39 (0.47–4.10)	0.510
LV GLS (%)	-13 (5)	-14 (5)	-12 (3)	-2 (-3–0)	0.116
LA strain (reservoir) (%)	15 (9)	16 (8)	9 (5)	**6 (4–9)**	**< 0.001**
LVDD					**< 0.001**
Grade I n, (%)	34 (34.0)	29 (38)	5 (21)	RR 0.51 (0.23–1.15)	0.081
Grade II n, (%)	30 (30)	26 (34)	4 (17)	RR 0.44 (2.12–8.59)	0.077
Grade III n, (%)	22 (22)	9 (12)	13 (54)	**RR 4.3 (2.1–8.6)**	**< 0.001**

Data are presented as median (IQR) or n (%), as appropriate

Abbreviations: HFmrEF, heart failure with mildly reduced ejection fraction; HFrEF, heart failure with reduced ejection fraction; IVST, interventricular septum thickness; LA, left atrial; LAVI, left atrial volume index; LV, left ventricular; LVDD, left ventricular diastolic dysfunction; LVEF, left ventricular ejection fraction; LVIDd, left ventricular internal diameter in diastole; GLS, global longitudinal strain

In an exploratory ordinal logistic regression, higher LAVI was seen associated with increasing LVDD classification, whereas no significant relationship was identified in ATTR genotype (Supplementary Materials, Table 2).

In a separate model including log-transformed NT-proBN; both NT-proBNP and ATTR genotype were associated with LVDD grade (Supplementary Materials, Table 3).

### LASr in diastolic function assessment with EACVI/ASE algorithm

Incorporating LASr into LVDD grading (available in 90% of total ATTR-CM), substituting for all TR velocity [8], resulted in an increase d proportion of patients classified with elevated indices of elevated LAP (68%) (Fig. 2). This was driven by reclassification of patients previously categorized as having undetermined LAP, with a corresponding reduction in this category to 3%. For the distribution of LVDD grading according to the different proposed approaches incorporating LASr, see Supplementary Figure S2.

### Morphological and functional variables between ATTR subgroups

Echocardiographic, morphological, and functional, variables are presented in Table 2. ATTRwt displayed significantly larger LAVI than ATTRv (46 vs. 37 mL/m², 95% CI − 13.3 to − 4.08; *p* = 0.001). Further, ATTRwt demonstrated lower cardiac output (4.0 vs. 5.2 L/min, 95% CI 0.07 to 1.36; *p* = 0.028) and lower LASr (9% vs. 16%, 95% CI 4 to 9; *p* < 0.001) when compared with ATTRv whereas LV EF (48% vs. 52%, 95% CI 0 to 8; *p* = 0.074) and LV GLS (-12% vs. -14%, 95% CI -3 to 0; *p* = 0.116) did not significantly differ between the groups.

Clinical variables between normal and elevated LAP groups (pooled LVDD grade II + III) can be viewed in Table 3.

**Table 3:** Clinical characteristics by LVDD classification

Variable	LVDD I normal LAP	LVDD II elevated LAP	Grade III restrictive filling	Combined grade II & III	Effect estimate	*p*
Female sex, n (%)	6 (17.6)	15 (46.9)	3 (15.0)	18 (34.6)	RR 2.0 (0.9–4.4)	0,139
Age, years	71 (15)	73 (10)	74 (11)	74 (11)	−3.0 (− 7.0– 0.1)	0,116
BSA, m²	1.94 (0.22)	1.76 (0.30)	1.88 (0.32)	1.82 (0.29)	0.10 (0.02–0.21)	**0.01**
Essential hypertension, n (%)	12 (35.3)	7 (23.0)	8 (36.0)	15 (28.8)	RR 0.8 (0.4–1.5)	0.635
Type 2 diabetes, n (%)	1 (3.0)	1 (3.0)	3 (14.0)	4 (7.7)	RR 2.6 (0.3–22.4)	0.644
Pacemaker, n (%)	1 (2.9)	6 (15.6)	3 (15.0)	9 (15.4)	RR 5.2 (0.7–40.0)	0.080
Paroxysmal AF, n (%)	5 (14.7)	7 (21.9)	12 (55.0)	19 (34.6)	RR 2.4 (1.0–5.7)	**0**.**049**
CHD, n (%)	6 (17.6)	4 (9.4)	6 (30.0)	10 (17.3)	RR 0.82 (0.44–1.53)	1.00
NT-proBNP, µg/L	557 (947)	1824 (3347)	1794 (2046)	1824 (2382)	−1089 (− 1729−598)	**< 0**.**001**

Data are presented as median (IQR) or n (%), as appropriate

Abbreviations: AF, atrial fibrillation; BSA, body surface area; CHD, coronary heart disease

Patients with elevated LAP displayed lower BSA (1.82 vs. 1.94 m^2^, 95% CI 0.02 to 0.21; *p* = 0.01), higher NT-proBNP (1824 vs. 557 µg/L, 95% CI − 1729 to − 598; *p* < 0.001) and higher prevalence of pxAF (*n* = 19 vs. 1, 95% CI 1.0 to 5.7; *p* = 0.049) when compared with normal LVDD grade I/LAP. When echocardiographic morphological and functional variables were compared (Table 4), those with elevated LAP displayed higher IVST (18 vs. 16 mm, 95% CI -3 to 1.0; *p* = 0.001) ,, LV mass (282 vs. 234 g, 95% CI -45 to -75; *p* = 0.006), LA volume (44 vs. 29 mL/m^2^, 95% CI -18.5 to -11.7; *p* < 0.001) as well as lower LASr (12 vs. 17%, 95% CI 1 to 7; *p* = 0.007) when compared with LVDD grade I/normal LAP. No significant differences in LV EF, GLS or cardiac output were seen between these two groups.

**Table 4:** Echocardiographic parameters by LVDD classification

Variable	LVDD I normal LAP	LVDD II elevated LAP	Grade III restrictive filling	Combined grade II & III	Effect estimate	*p*
Lvidd, mm	43 (7)	43 (7)	44 (8)	43 (8)	-1.0 (-3.0 to 1.0)	0.323
IVST, mm	16 (3)	18 (5)	19 (2)	18 (4)	-2.0 (-3.0 to -1.0)	**0.001**
LV mass, g	234 (78)	279 (77)	302 (94)	282 (95)	-45.0 (-75.0 to -13.0)	0.006
LAVI, mL/m^2^	29 (7)	43 (9)	49 (10)	44 (12)	-15.0 (-18.5 to -11.7)	**< 0.001**
Cardiac output, L/min	5.1 (1.9)	5.3 (2.4)	4.1 (1.5)	5.0 (1.9)	0.1 (− 0.5–0.8)	0.611
LVEF, %	51 (11)	51 (12)	50 (14)	51 (12)	1.0 (− 3.0–5.0)	0.757
Normal (≥ 50%), n (%)	20 (59)	19 (61)	11 (50)	30 (57.7)	RR 0.95 (0.66–1.36)	0.824
HFmrEF (41–49%), n (%)	11 (32)	10 (32)	6 (27)	16 (31.0)	RR 0.92 (0.49–1.74)	0.815
HFrEF (≤ 40%), n (%)	2 (6)	2 (6)	5 (20)	6 (12.0)	RR 1.9 (0.41–8.9)	0.475
LV GLS, %	−14 (5)	−13 (4)	−11 (3)	−12 (4)	−2.0 (− 3.0–4.2)	0.103
LASr, %	17 (9)	16 (10)	9 (6)	12 (9)	4.0 (1.0–7.0)	**0**.**007**

Data are presented as median (IQR) or n (%), as appropriate

Abbreviations: HFrEF, heart failure with reduced ejection fraction; HFmrEF, heart failure with mildly reduced ejection fraction; IVST, interventricular septum thickness; LA, left atrial; LASr, left atrial reservoir strain; LV, left ventricular; LV GLS, left ventricular global longitudinal strain; LV mass, left ventricular mass; LVIDd, left ventricular internal diameter in diastole; LVEF, left ventricular ejection fraction; LAVI, left atrial volume index

## Discussion

We graded LV diastolic dysfunction in a well-characterized ATTR-CM cohort using the 2016 ASE/EACVI algorithm [10], including both ATTRv and ATTRwt. We specifically aimed at assessing prevalence of elevated LAP, by using the conventional algorithm and by adding LASr. While 53% of our patients displayed elevated LAP, only 22% displayed indices of restrictive filling. ATTRwt displayed a higher frequency of restrictive filling than ATTRv, highlighting genotype-specific differences in diastolic profiles, although a high proportion of paroxysmal AF in this specific group certainly questions the specificity. Incorporation of LASr to diastolic assessment does not alter prevalence of restrictive filling but raised prevalence of elevated filling pressure from 53 to 68% in the overall study group and reduced indeterminate cases from 13 to 3%.

Cardiac amyloidosis, irrespective of type, is often associated with restrictive hemodynamics [11, 12]. Previous studies have reported echocardiographic indices of elevated LAP and restrictive filling as common in CA [6, 13, 14] with prevalence varying by subtype [5, 15]. Several red flags are described in ATTR-CM, and restrictive filling pattern by Doppler is frequently cited and endorsed by current recommendations [11, 12, 16]. In this study, we report only a minority with restrictive filling despite elevated filling pressures being common. Overt emphasis on restrictive filling alone as a marker may be misleading and result in overlooking those with more preserved diastolic function. Notably, patients with elevated LAP exhibited clinical signs suggestive of more advanced disease, including high NT-ProBNP levels, increased IVST, and greater LV mass compared to those with normal LAP.

### Genotype-specific diastolic status

Another key finding is the markedly higher prevalence of restrictive filling in ATTRwt when compared with ATTRv in this study. Contrary to our findings, Oghina et al. [6] reported a higher prevalence of LVDD grade III in ATTRv patients. This discrepancy may reflect differences in cohort composition, such as inclusion of paroxysmal AF patients and differences in genotype, as our study mainly included ATTR Val30Met. Additionally, our cohort was evaluated shortly after diagnosis, aiming to capture early disease stages.

ATTRv and ATTRwt displayed different distribution of diastolic function status when compared as two subgroups, with greater prevalence of restrictive filling pattern in the latter. Despite representing a minority of the study population, ATTRwt patients comprised over half of all cases with restrictive filling. This subgroup was significantly older, displayed a higher prevalence of previous AF and coronary disease, larger LA volume, lower LASr and lower cardiac output. Despite similar LVEF, LV GLS and myocardial thickness between groups, the higher prevalence of restrictive filling in ATTRwt may reflect a combination of age-related comorbidities and hemodynamic burden beyond amyloid infiltration.

Although ATTRv and ATTRwt share common pathophysiology, their clinical expressions differ. ATTRwt is characterized by late onset, a predominance of cardiac involvement, and a higher likelihood of delayed diagnosis [17]. In contrast, ATTR V30M patients are often identified earlier due to neurological manifestations and greater disease awareness, aiding early disease detection [18].Taken together, our results underscore that ATTR-CM is a heterogenous condition, making a uniform definition of echocardiographic red flags or hemodynamic profiles challenging. Changes in genotype effects after adjustment point to different underlying disease mechanisms. Adjustment for NT-proBNP, a marker of current hemodynamic stress that is not incorporated into diastolic grading, preserved the association between genotype and LVDD severity. In contrast, adjustment for LAVI, an integral component of diastolic grading and a marker of chronic atrial remodeling, attenuated genotype effects, consistent with overadjustment for structural changes secondary to ATTR-CM. Overall, these findings support the interpretation that more advanced atrial remodeling, potentially reflecting atrial amyloid involvement [19], explains the higher prevalence of restrictive filling observed in ATTRwt.

However, given the small study population of ATTRwt together with the exploratory nature of these analyses and partial violation of model assumptions, these observations should be interpreted with caution.

### The use of left atrial strain and diastolic dysfunction

Using LASr in addition to the 2016 ASE/EACVI diastolic algorithm has been shown to improve the accuracy of diastolic function assessment [8]. In our cohort, incorporating LASr increased the feasibility of diastolic grading and proportion of patients identified with elevated LAP. Recent evidence suggests that atrial amyloid infiltration directly impacts LA function [20]. Beyond its role as a predictor of mortality [21] and possible marker for elevated LAP in the general population [8, 22, 23], LASr has been proposed to hold specific diagnostic value in ATTR-CM [24, 25]. Furthermore, strain is used to evaluate LA systolic dysfunction in immunoglobulin light chain (AL) amyloidosis [26] and could possibly differentiate amyloid involvement over other underlying causes of diastolic dysfunction [27].

In our cohort, LASr was markedly reduced and significantly lower in the group with elevated LAP compared to normal LAP, in line with previous reports [20, 25]. This supports the concept that amyloid infiltration compromises LA contractility and thereby alters LV filling properties. Reduced LASr in CA may therefore reflect not only LV myocardial involvement but also LA amyloid burden. We propose that incorporating LASr has the potential to unmask a greater prevalence of elevated filling pressures in ATTR-CM.

### Clinical implications

Since echocardiography is widely utilized to screen and raise suspicion of CA before advanced testing [15], it is essential to recognize the range of expected hemodynamic profiles and to interpret these patterns in light of patient management. While restrictive physiology is traditionally considered a hallmark of CA, our findings suggest it is uncommon in early stages and varies by genotype. This has direct implications for clinical practice, as reliance on restrictive filling as a red flag may lead to under-recognition of CA in patients with more preserved diastolic function.

Further studies are also required to address LASr’s role in ATTR-CM, and to assess if reduced strain primarily reflects increased LAP, atrial amyloid burden, or both. Clinically, this supports integrating atrial strain into routine assessment to better characterize disease stage, guide therapy, and predict outcomes. As therapeutic options expand, timely recognition of subtle hemodynamic abnormalities becomes increasingly important to initiate treatment before advanced heart failure develops.

Non-invasive evaluation of diastolic function remains challenging in ATTR-CM, given the high prevalence of atrial fibrillation that complicates interpretation of standard indices [28]. Future directions should explore hemodynamic evaluation during provocation, such as submaximal exercise during TTE, as this might unmask higher grades of LVDD [29].

### Limitations

Small study populations are a recurring limitation in CA research given the rarity of the condition, yet our findings offer meaningful value to existing data. The retrospective design limited the availability of certain echocardiographic parameters for comprehensive evaluation of diastolic function. Furthermore, our study did not include patients with persistent AF, which is common in ATTR-patients, and this limits generalizability of our findings. Finally, although DPD-scintigraphy was not available in seven patients, all diagnoses were established by experienced amyloidosis specialists, and we are confident about the accuracy of case classification [30]. LASr and LAVI both reflect atrial remodeling, and LASr incorporation may therefore influence LVDD classification independently of filling pressures. Moreover, although multiple LASr-adapted strategies were explored, patient-level reclassification and agreement analyses were not performed and represent an important area for future investigation.

## Conclusion

Elevated filling pressures are common in ATTR-CM, but restrictive filling is infrequent and varies by genotype, being more prevalent in ATTRwt. Incorporation of LASr into diastolic assessment improves identification of elevated LAP and reduces indeterminate cases. These findings emphasize the heterogeneity of ATTR-CM and the need for genotype-specific, personalized echocardiographic evaluation.

## Supplementary Information

Below is the link to the electronic supplementary material.

Supplementary Material 1

## Data Availability

The data underlying this article cannot be shared publicly due to the privacy regulations and requirements of authorities. The data will be shared at reasonable requests to the corresponding author.
