# Supplementary material for: Elevated filling pressures but rare restrictive physiology in transthyretin amyloid cardiomyopathy: genotype-specific patterns and the role of left atrial strain
Source: Int J Cardiovasc Imaging. 2026 Mar 4;42(7):1325–33. doi: 10.1007/s10554-026-03667-z (PMC13375967; doi:10.1007/s10554-026-03667-z)
Supplement: Supplementary file 1 — Supplementary Material 1 [file 10554_2026_3667_MOESM1_ESM.docx]

Supplementary Materials


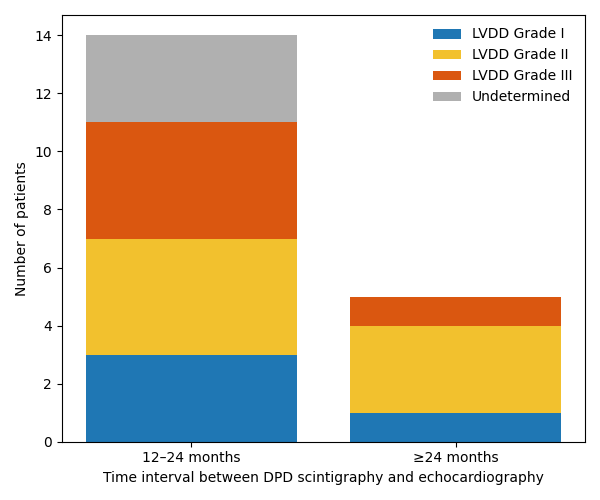


Figure S1 Distribution of LVDD grades according to time interval between DPD scintigraphy and echocardiography. The relative distribution of LVDD grades was similar across time intervals, with no apparent shift toward more advanced diastolic dysfunction at longer intervals.

Supplementary Table S1. Distribution of abnormal diastolic criteria by LVDD grade and genotype

| Criterion  (Abnormal*) | ATTRv (n = 76 ) | | | ATTRwt | | | Availability  N, (%) |
| --- | --- | --- | --- | --- | --- | --- | --- |
|  | LVDD I  (n = 29) | LVDD II  (n = 26) | LVDD III  (n = 9) | LVDD I  (n = 5) | LVDD II  (n = 4) | LVDD III  (n = 13) |  |
| **E/e′ >14 (missing)** | 3 (7) | 21 (5) | 4 (3) | 0 (1) | 1 (1) | 5 (5) | 74 |
| **LAVI >34 ml/m²**  **(missing)** | 5 (2) | 23 (2) | 4 (5) | 2 (1) | 4 (4) | 8 (3) | 70 |
| **TRV >2.8 m/s (missing)** | 1 (8) | 6 (8) | 4 (4) | 1 (0) | 3 (0) | 8 (1) | 70 |
| **LASr <18% (missing)** | 13 (4) | 16 (0) | 5 (4) | 3 (1) | 4 (0) | 11 (1) | 90 |

*Abnormal by ASE/EACVI 2016

Supplementary Table S2. Ordinal logistic regression for LVDD grade (with LAVI)

| Predictor | Odds Ratio (OR) | 95% CI | p-value |
| --- | --- | --- | --- |
| ATTR genotype (ATTRwt vs ATTRv) | 0.36 | 0.10–1.33 | 0.125 |
| Left atrial volume index (per ml/m²) | 1.18 | 1.10–1.27 | <0.001 |
| Age (per year) | 1.01 | 0.95–1.08 | 0.680 |
| Female sex | 1.10 | 0.36–3.37 | 0.874 |
| Paroxysmal AF | 0.57 | 0.16–2.07 | 0.394 |

- N = 72
- Likelihood ratio χ² p < 0.001
- Nagelkerke R² = 0.55

Supplementary Table S3. Ordinal logistic regression for LVDD grade (with log(NT-ProBNP)

| Predictor | Odds Ratio (OR) | 95% CI | p-value |
| --- | --- | --- | --- |
| ATTR genotype (ATTRwt vs ATTRv) | 3.2 | 1.1–9.5 | 0.041 |
| log(NT-proBNP) | 1.93 | 1.29–2.88 | 0.001 |
| Age (per year) | 1.01 | 0.96–1.06 | 0.724 |
| Female sex | 0.97 | 0.37–2.58 | 0.956 |
| Paroxysmal AF | 0.58 | 0.20–1.69 | 0.317 |

- N = 83
- Likelihood ratio χ² p < 0.001
- Nagelkerke R² = 0.32


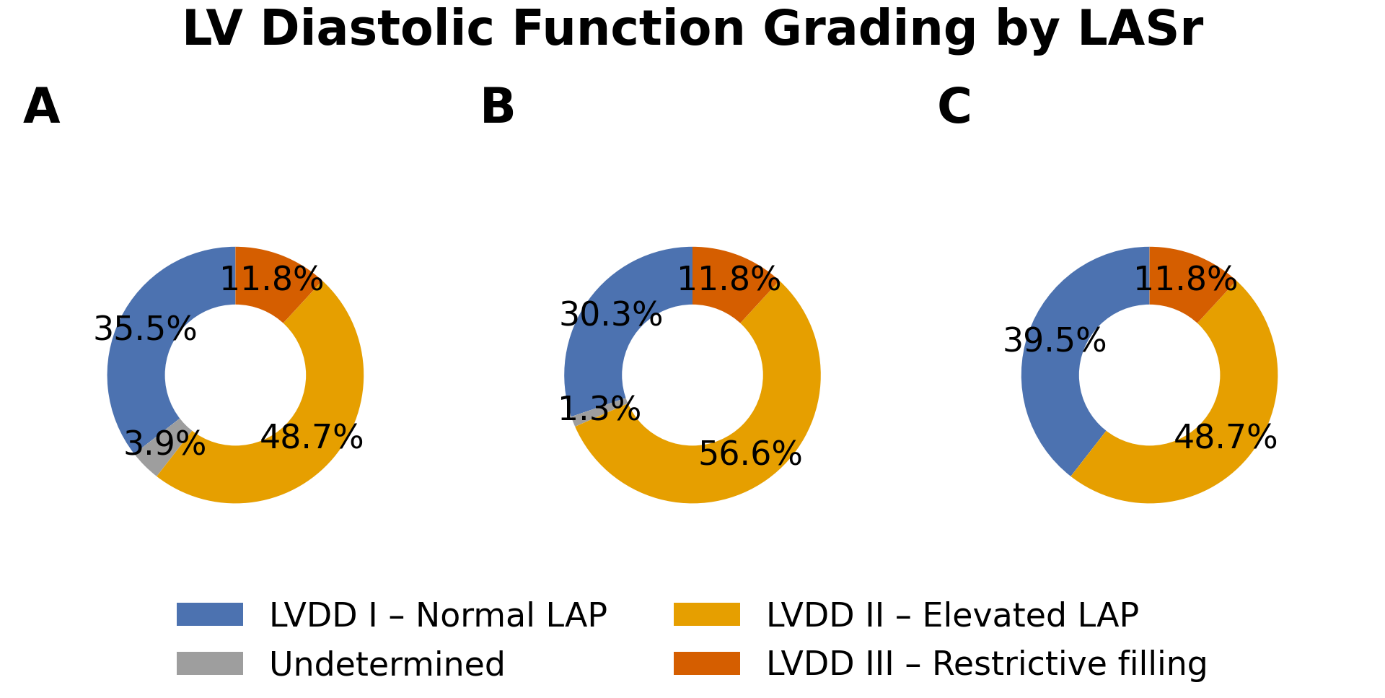


Figure S2. Left Ventricular Diastolic Dysfunction grading by 2016 ASA/EACVI and **A)** Substitute TR velocity with LASr <18%, **B)** Add LASr <18% to additional criteria and **C)** Substitute any missing additional criteria with LASr <18%.
